# Identification of brassinosteroid genes in *Brachypodium distachyon*

**DOI:** 10.1186/s12870-016-0965-3

**Published:** 2017-01-06

**Authors:** Claudia Corvalán, Sunghwa Choe

**Affiliations:** 1 School of Biological Sciences, College of Natural Sciences, Seoul National University, Seoul, 08826 South Korea; 2 Convergence Research Lab for Plant Functional Products, Advanced Institutes of Convergence Technology, Suwon, 16229 Gyeonggi-do South Korea; 3 Plant Genomics and Breeding Institute, Seoul National University, Seoul, 08826 South Korea

**Keywords:** BIN2, *Brachypodium distachyon*, Brassinosteroids, BRI1, DWF4, Monocots, Propiconazole

## Abstract

**Background:**

Brassinosteroids (BRs) are steroidal phytohormones that are involved in diverse physiological processes and affect many important traits, such as plant stature, stress tolerance, leaf angle, fertility, and grain filling. BR signaling and biosynthetic pathways have been studied in various plants, such as the model dicot *Arabidopsis thaliana*; however, relatively little is known about these pathways in monocots.

**Results:**

To characterize BR-related processes in the model grass *Brachypodium distachyon*, we studied the response of these plants to the specific BR biosynthesis inhibitor, propiconazole (Pcz). We found that treatments with Pcz produced a dwarf phenotype in *B. distachyon* seedlings, similar to that observed in Pcz-treated Arabidopsis plants and in characterized BR-deficient mutants. Through bioinformatics analysis, we identified a list of putative homologs of genes known to be involved in BR biosynthesis and signaling in Arabidopsis, such as *DWF4*, *BR6OX2*, *CPD*, *BRI1*, and *BIN2*. Evaluating the response of these genes to Pcz treatments revealed that candidates for *BdDWF4*, *BR6OX2* and, *CPD* were under feedback regulation. In addition, Arabidopsis plants heterologously expressing *BdDWF4* displayed tall statures and elongated petioles, as would be expected in plants with elevated levels of BRs. Moreover, heterologous expression of *BdBIN2* in Arabidopsis resulted in dwarfism, suggesting that BdBIN2 functions as a negative regulator of BR signaling. However, the dwarf phenotypes of Arabidopsis *bri1-5*, a weak *BRI1* mutant allele, were not complemented by overexpression of *BdBRI1*, indicating that BdBRI1 and BRI1 are not functionally equivalent.

**Conclusion:**

We identified components of the BR biosynthetic and signaling pathways in Brachypodium, and provided examples of both similarities and differences in the BR biology of these two plants. Our results suggest a framework for understanding BR biology in monocot crop plants such as *Zea mays* (maize) and *Oryza sativa* (rice).

**Electronic supplementary material:**

The online version of this article (doi:10.1186/s12870-016-0965-3) contains supplementary material, which is available to authorized users.

## Background

Brassinosteroids (BRs) are plant polyhydroxylated steroids that function as growth-promoting hormones. They have been implicated in many developmental and physiological processes in *Arabidopsis thaliana*, including vascular differentiation, stem and root elongation, reproductive development, photomorphogenesis, and stress responses. Hence, mutant plants defective in BR synthesis or perception display characteristic phenotypes such us short stature, round, curled leaves, short petioles, and reduce fertility [1–3].

The BR-biosynthetic and signaling pathways have been well studied in *Arabidopsis thaliana*. A family of enzymes belonging to Cytochrome P450s mediates most of the steps of BR biosynthesis, and characterization of mutants defective in these enzymes contributed to the understanding of BR biology [4, 5]. Various approaches have established the components and mechanisms of the BR signaling pathway; once BR binds to the receptor kinase, BRASSINOSTEROID-INSENSITIVE 1 (BRI1), transmits the signal to downstream genes, eventually leading to the repression or activation of BR responsive genes [6, 7].

It appears that BR signaling is conserved between monocotyledonous and dicotyledonous plants, as some counterparts of the Arabidopsis proteins are present in *Oryza sativa* (rice). For instance, orthologs of the receptor BRI1 and co-receptor BRI1-ASSOCIATED RECEPTOR KINASE 1 (BAK1), named OsBRI1 and OsBAK1, respectively; two homologs of the negative regulator of the BR signaling protein BR INSENSITIVE 2 (BIN2), GSK3/SHAGGY-like kinase 1 and 2 (OsGSK1 and OsGSK2); and an ortholog of a major transcription factor in the BR transduction pathway BRASSINAZOLE RESISTANT 1 (OsBZR1) have been identified in rice [7–10].

Despite the similarities with dicot BR biology, some differences have been noted in monocots too. Brassinolide (BL), the most active form of BR and end product of BR synthesis in Arabidopsis, has not been detected in rice, where apparently castasterone (CS) seems to be the end product. Furthermore, no homolog of CYP85A2, which mediates BL synthesis, has been found in rice [11]. In addition, components of BR signaling with no known orthologs in Arabidopsis have been identified in rice, indicating the existence of specific BR functions in monocots or some degree of functional redundancy. Examples of these components are DWARF AND LOW-TILLERING (DTL) and TILLER ANGLE INCREASED CONTROLLER (LIC), which act downstream of OsBRI1 and OsGSK2 to positively and negatively regulate rice BR signaling, respectively [12, 13], and the U3 ubiquitin ligase TAIHU DWARF 1 (TUD1), which interacts genetically and physically with D1/OsRGA, a heterotrimeric G protein subunit involved in gibberellin (GA) and BR responses [14].

*Brachypodium distachyon* (hereafter Brachypodium) is a relatively new model plant proposed for the study of grasses, since it has a short live cycle, is self-fertile, easy to grow, and is more closely related to Poaceae than is *A. thaliana* [15]. However, little is known about phytohormones in *B. distachyon*. Only two dwarf mutants, with defects in the BRI1 receptor and a C-6 oxidase (BRASSINOSTEROID DEFICIENT DWARF 1; BRD1), have been characterized in this model plant [16, 17].

In the present work, we used a specific BR biosynthesis inhibitor drug, propiconazole (Pcz) [18], to study BR action in Brachypodium and characterize orthologs of the BR-biosynthetic enzymes DWARF4, BR6ox2, and CPD. Furthermore, by heterologous complementation, we studied homologs of two important genes in the BR signaling pathway, the receptor BRI1 and the negative regulator BIN2. This work revealed important similarities and differences between the BR synthesis and signaling pathways in Arabidopsis and Brachypodium.

## Results

### *Brachypodium distachyon* seedlings display BR-related phenotypes in response to propiconazole treatment

**Fig. 1:**
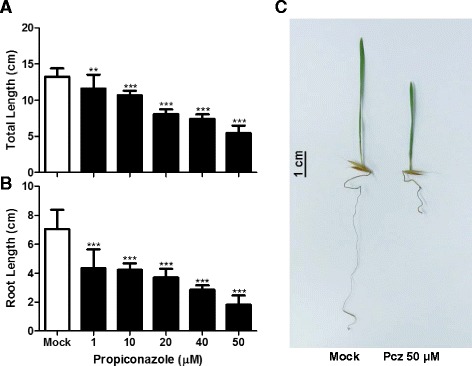
Brachypodium seedlings exhibit dose-dependent dwarfism in response to propiconazole treatment. **a** Total lengths of seedlings and (**b**) roots after 7 days of exposure to 0 (Mock) to 50 μM Pcz. **c** Morphology of 7-days-old seedlings subjected to the mock treatment and the strongest Pcz concentration tested. The graphs represent average value (*n* > 10) and error bars standard deviation. Significant differences among treatments were determined by Student’s *t*-test. **, *P* < 0.001 and ***, *P* < 0.0001

**Fig. 2:**
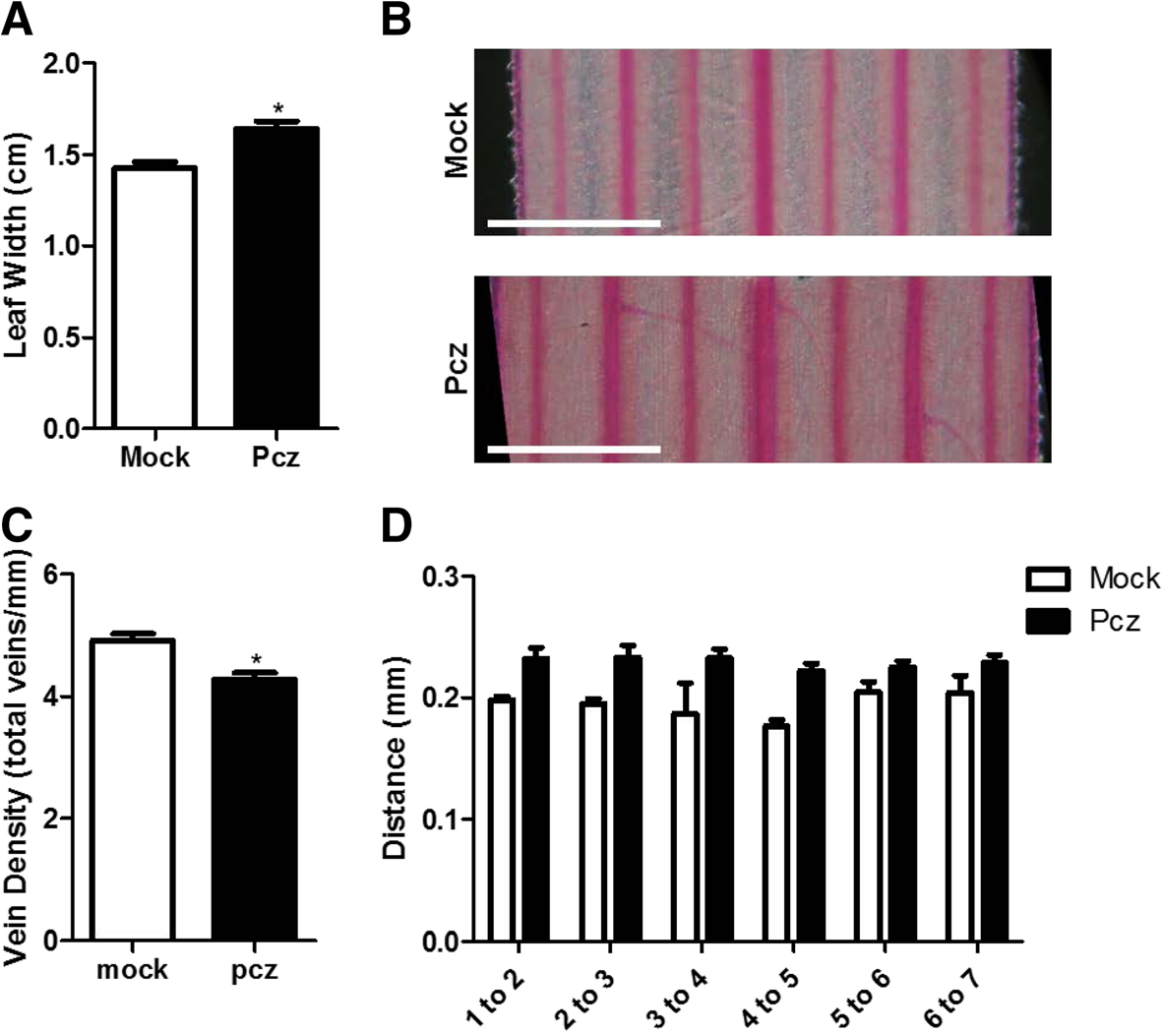
Brachypodium leaf width and distance between veins are affected by Pcz. **a** Leaves of seedlings undergoing mock or Pcz (50 μM) treatments were measured and dissected in the middle. **b** Safranin O-stained leaf sections were used to count and observe veins under the microscope. **c** The vein density values were obtained by dividing the total number of veins in the section by the width of the leaf. **d** The distance between veins was measured using ImageJ software. Scale bar = 0.5 mm. The graphs represent average value (*n* > 5) and error bars standard deviation. Significant differences among treatments were determined by Student’s *t*-test. *, *P* < 0.05

Since Brachypodium is a relatively new model plant, studies of processes and genes regulated by phytohormones in this organism are limited. The unavailability of Brachypodium BR-defective or -insensitive mutants made it challenging to determine if BR function is conserved across plant species. To study the mode of action of BRs and the factors involved in these processes, we first treated Brachypodium Bd21 seedlings with the BR-specific inhibitor Pcz in concentrations ranging from 1 to 50 μM for 7 days (Fig. 1A-C). We observed a dose-response reduction of the total lengths of plants; 1 μM Pcz resulted in a ~13% reduction in length compared to mock conditions, whereas 50 μM, the greatest concentration tested, resulted in a ~60% reduction (Fig. 1A). This reduction was especially severe in roots; the main root was reduced by 50% in plants treated with 20 μM Pcz relative to control plants, and by over 74% in those treated with 50 μM (Fig. 1B). The Pcz-induced inhibitory effects were observed under both light and dark conditions (Additional file 1: Figure S1). In contrast to the effect on the overall length of the plant, leaf length was only reduced by ~25% under the strongest Pcz treatment (Additional file 2: Figure S2). To evaluate if Pcz had other effects on leaf morphology or the vascular system, we examined the leaf architecture in more detail, focusing on venation patterns, total number of veins, vein density, and distance between veins. Interestingly, we found that Pcz-treated leaves were thicker and wider than those from plants grown under control conditions, but that the number of veins remained the same. As a consequence, the distance between veins is on average greater in the treated leaves, so vein density is reduced by Pcz treatment (Fig. 2).

### Identification of genes modulating BR response in Brachypodium

**Fig. 3:**
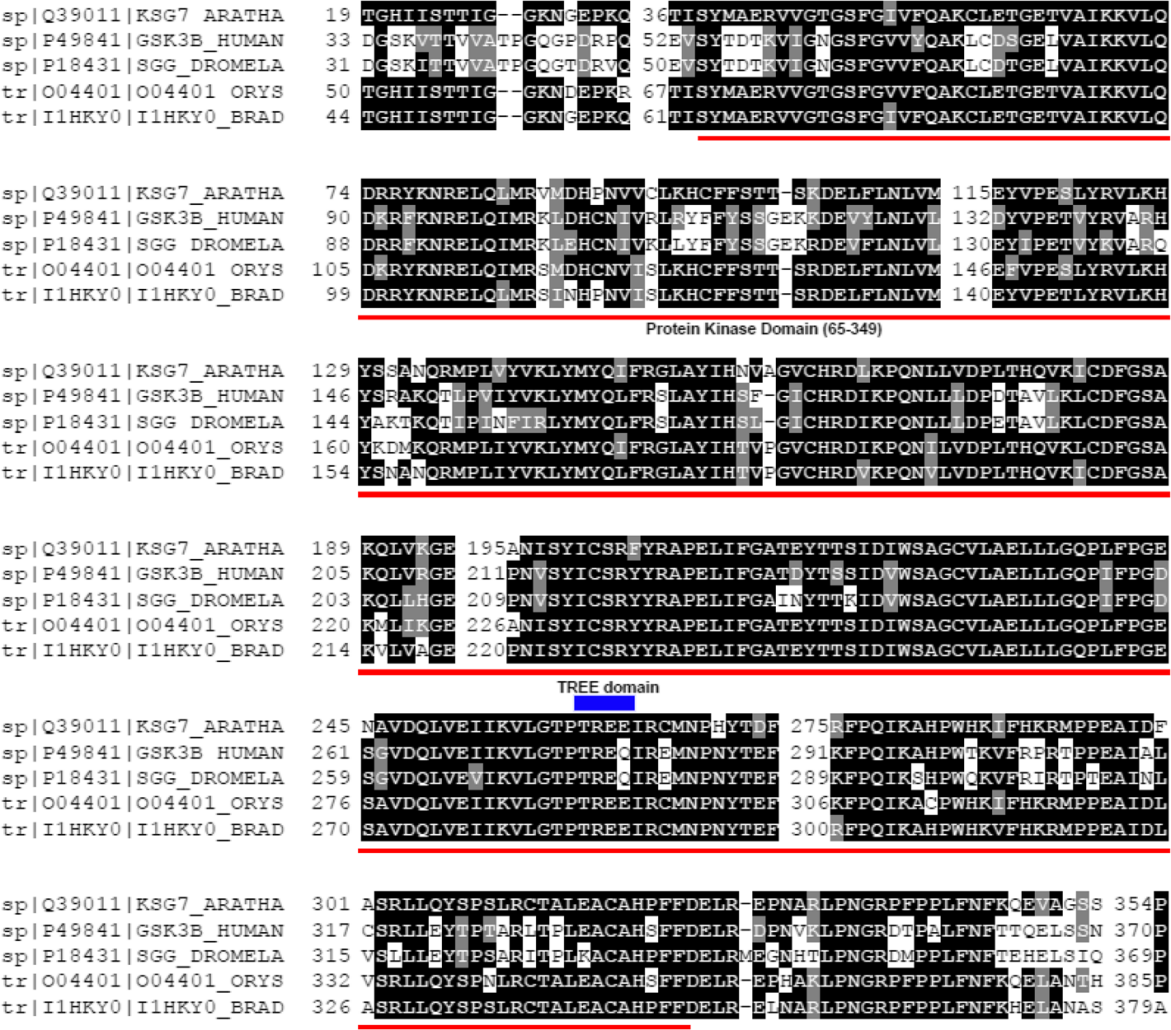
BdBIN2 shows conserved motifs with other GSK3/SHAGGY-like kinase proteins. Part of the multiple sequence alignment (MSA) of the BIN2 ortholog in Brachypodium (1HKY0) with AtBIN2 (Q39011) and a GSK3 homolog from human (P49841) and *D. melanogaster* (P18431). The conserved protein kinase domain is underlined in red and the TREE domain, which is well conserved in plants, is represented by a blue rectangle

With the release of the whole genome sequence of *Brachypodium distachyon*, we were able to search for homologs that could participate in brassinosteroid biosynthesis or signaling in this species. We conducted a BLAST search of the Brachypodium database using the amino acid sequences of Arabidopsis proteins and then used multiple sequence alignment (MSA) and phylogenetic analysis to reduce the number of homolog candidates to one per protein for further evaluation. In the case of BIN2, the MSA and phylogenetic analysis were performed using Arabidopsis BIN2, BIN2-LIKE 1 (BIL1), and BIN2-LIKE 2 (BIL2) and also the rice homologs OsGSK1, OsGSK2, and OsSKetha. We also performed a second MSA, including *A. thaliana* BIN2, a GSK3 from human (P49841) and *D. melanogaster* (P18431), and rice *OsKetha* (Y13437) using the T-Coffee program to screen for conserved motifs. We observed that the kinase domain and the TREE domain, identified as being a putative Thr phosphorylation site by caseine kinase II and thus important for negative regulatory events, were also present in the Brachypodium homolog (Fig. 3). Thus, we selected *Bradi2g48280*, *Bradi2g32620*, *Bradi2g36370*, *Bradi1g23550*, *Bradi1g69040*, *Bradi4g43110*, and *Bradi1g15030* as homologs of *BRI1*, *BIN2*, *BSU1*, *BZR1*, *DWF4*, *CPD*, and *BR6ox2*, respectively, for further characterization.

### Brachypodium *DWF4*, *CPD*, and *BR6OX2* homologs are under feedback regulation

**Fig. 4:**
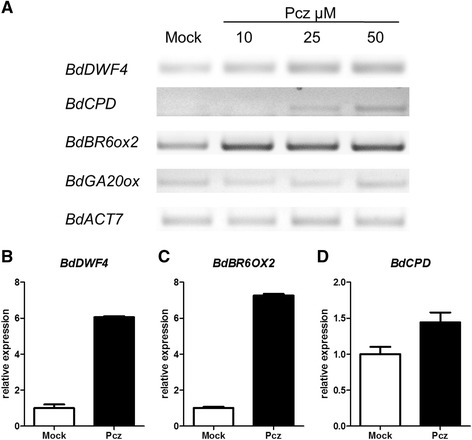
Feedback regulation of Brachypodium BR biosynthetic genes after Pcz treatments. **a** Semi-quantitative RT-PCR of putative BR biosynthetic genes *BdDWF4*, *BdBR6OX2*, and *BdCPD* and the GA biosynthetic gene *BdGA20ox* after treatment with the indicated concentrations of Pcz and a mock control. *BdACT7* is used as an internal loading control. **b**-**d** Quantitative real-time RT-PCR analysis evaluating the expression of the same genes under just mock or 50 μM Pcz

To verify that the genes identified by bioinformatics tools were in effect orthologs of BR biosynthetic genes in *B. distachyon*, we examined their transcripts levels in seedlings subjected to various Pcz treatments. To obtain a general sense of the gene expression response to the treatments, we performed semi-quantitative RT-PCR analysis on seedlings treated with three different concentrations of Pcz (10, 25, and 50 μM) along with mock. We noticed that the transcript levels of the three BR-related genes increased with increasing Pcz concentrations. The GA biosynthesis gene *GA20ox1* was used as a control for the Pcz treatment, to ensure that only BR-related genes were inhibited by the treatment (Fig. 4A). After determining the conditions that produced a clear transcriptional response, we carried out a similar experiment using just mock and one Pcz concentration (50 μM) to analyze *BdDWF4*, *BdBR6ox2*, and *BdCPD* mRNA levels by quantitative real-time RT-PCR. For the first two genes, Pcz-treated seedlings exhibited a more than six-fold increase in expression with respect to the mock treatment, while *BdCPD* was only slightly upregulated (Fig. 4B-D). Nonetheless all three candidate genes were found to be under negative feedback regulation, a characteristic of genes involved in BR biosynthesis. Normally, the expression of these genes is inhibited by the presence of BR and enhanced when BR levels are low such as under treatment with inhibitors (i.e., a negative feedback mechanism regulates the activity of genes downstream of BR). Thus, *Bradi1g69040* (*BdDWF4*), *Bradi4g43110* (*BdCPD*), and *Bradi1g15030* (*BR6ox2*) may indeed be involved in BR biosynthesis in *Brachypodium distachyon*.

### Overexpression of *BdDWF4* in *Arabidopsis thaliana* produce long and slender plants

**Fig. 5:**
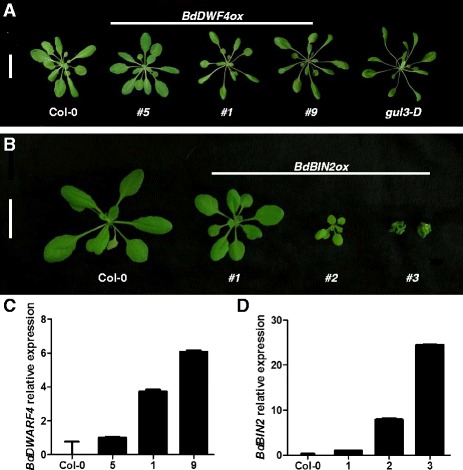
Phenotypes of the *BdDWF4ox* and *BdBIN2ox* transgenic lines, suggesting the involvement of *BdDWF4* in BR biosynthesis and *BdBIN2* in responses, respectively. **a** Morphology of three independent *BdDWF4* overexpression lines (#5, #1, and #9) along with wild type Col-0 and *gul3*-*D* BR overproducing mutant. **b** Phenotypes of the wild type Col-0 and *BdBIN2* overexpressing lines. Line #2 represent mild phenotype, while #3 are examples of severe dwarfism. Scale bar = 2 cms. **c**-**d** Quantitative real-time analysis evaluating the heterologous expression of *BdDWF4* and *BdBIN2* respectively. Line #5 **c** and #1 (**d**) which don’t show distinctive phenotype from wild type were used to compare expression of the rest of the lines. A*tUBQ10* was used as internal loading control

To further evaluate the involvement of *BdDWF4* in BR responses, we introduced the full-length coding sequence of this gene into *A. thaliana* under the CaMV 35 s promoter. From a total of 26 analyzed *BdDWF4* overexpressing lines (*BdDWF4ox*), half displayed an increase in plant height and had longer and narrower leaves than the wild-type control, Col-0 (Fig. 5A). These characteristics are typical of brassinosteroid-overproducing mutant plants, such as *gulliver3 - D* (*gul3*-*D*), in which the increase in BR level is the result of activation tagging of *DWF4*. Analysis of the expression level in transgenic plants corroborated that degree of the phenotype is proportional to the levels of transcripts (Fig. 5C). The results strongly suggest that *Bradi1g69040* plays a significant role in *Brachypodium* growth and development, probably by functioning in the BR biosynthetic pathway.

### Overexpression of *BdBIN2* in Arabidopsis results in a stunted growth phenotype

We then overexpressed a candidate gene involved in BR signaling *BdBIN2*, in *A. thaliana* to study the effect of this gene on the phenotypes of the transgenic plants. From a total of 32 analyzed lines, nearly 14% of the transgenic plants presented severe dwarf phenotypes, were unable to set flowers, and died after a few weeks (Fig. 5B). These extreme dwarf plants presented the highest level of transcript accumulation (Fig. 5D). Other *BdBIN2ox* lines showed milder phenotypes that were able to produce seeds but still exhibited a compact stature and smaller curled leaves compared with the wild type (Fig. 5B). These findings insinuate that *Bradi2g32620* is a functional ortholog of BRIN2 in Brachypodium that operates as a negative regulator of the BR signaling pathway.

### *BdBRI1* overexpression does not revert the weak phenotype of *A. thaliana bri1*-*5*

**Fig. 6:**
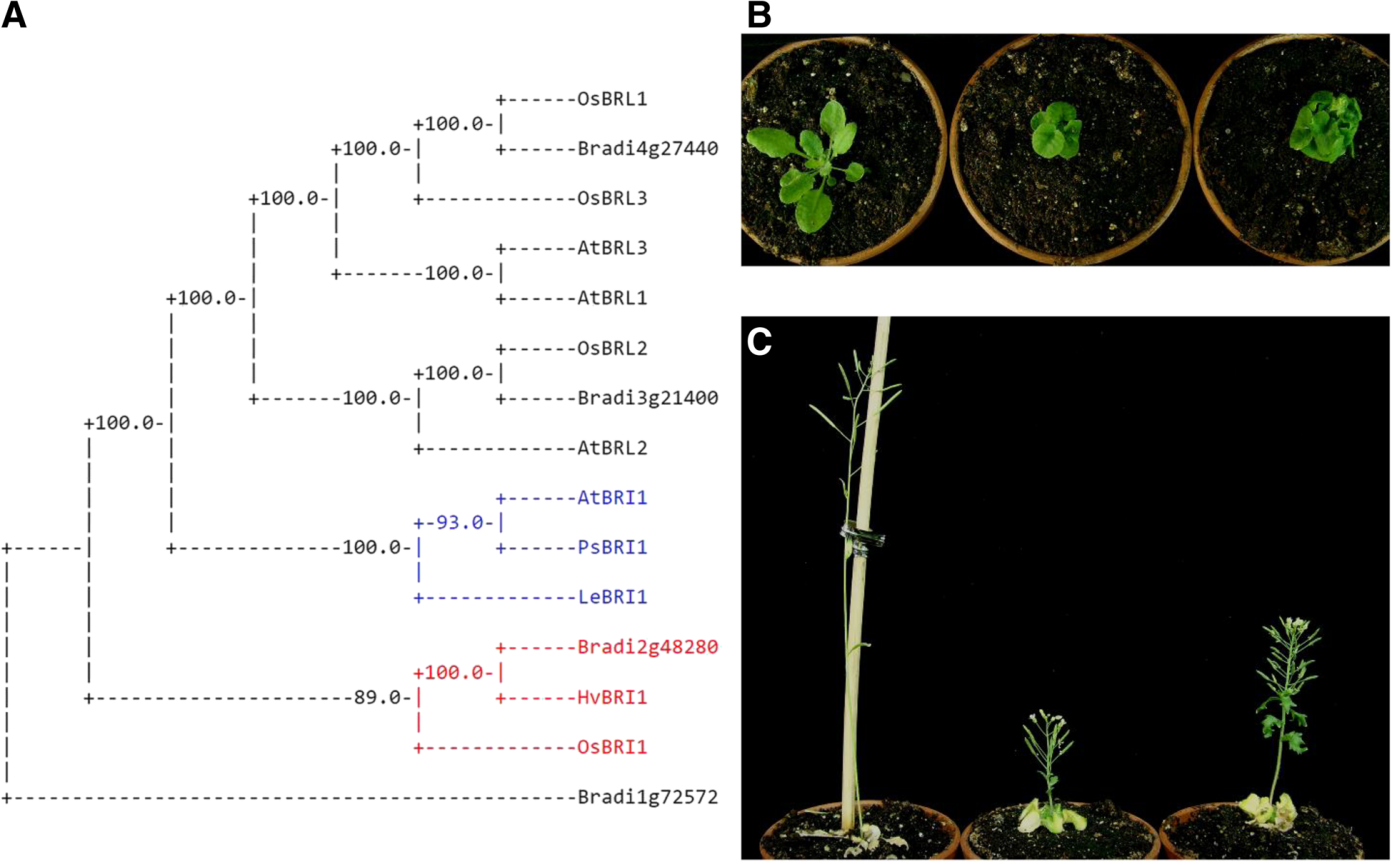
*BRI1* homolog in *Brachypodium* fails to rescue the phenotypes of the BR-insensitive mutant *bri1*-*5*. **a** Phylogenetic analysis showing Brachypodium BRI1 candidate proteins and BRI1 homologs in other species, including Arabidopsis, tomato, pea, rice, and barley. **b** Phenotypes of 3-week- or (**c**) 6-week-old WS-2 wild-type adult plants, the *bri1*-*5* mutant, and transgenic *bri1*-*5*/*BdBRI1ox* plants

A search for *BRI1* in the Brachypodium genome yielded three candidate genes (Additional file 3: Table S1) that we arranged in a phylogenetic tree along with other BRI1 proteins described in various dicot (*A. thaliana*, pea (*Pisum sativum*), and tomato (*Lycopersicon esculentum*)) and monocot (rice (*Oryza sativa*) and barley (*Hordeum vulgare*)) plants. As a reference, we also used BRI1-LIKE proteins BRL1, BRL2, and BRL3 from Arabidopsis and rice. As shown in Figure 6A, we observed that the protein product of *Bradi2g48280* was in the same group as orthologs of BRI1 in monocots, and although it shares only ~53% amino acid sequence identity with AtBRI1, it shares more than 80% identity with amino acid sequences in its monocot counterparts. To test if *Bradi2g48280* (named BdBRI1 from now on) has the same functions as AtBRI1, we performed heterologous complementation experiments. Specifically, we examined if overexpression of *BdBRI1* was able to revert the dwarf phenotype of the BR-insensitive *bri1*-*5* mutant, which is a weak allele of *AtBRI1*. In a first experiment we obtained 13 independent lines, all exhibiting dwarf phenotypes similar to *bri1*-*5*. Similarly, in a second transformation we obtained another 21 transgenic lines resistant to the Basta which didn’t show rescued phenotypes (Additional file 4: Figure S3A); *bri1*-*5/BdBRI1ox* lines were slightly taller than the *bri1*-*5* control, but were still dwarfed with abnormal leaf morphology (Fig. 6B-C). These results indicate that although *Bradi2g48280* is likely a homolog of *Arabidopsis thaliana BRI1*, these two genes may have different structure and/or functions that make BdBRI1 incapable of operating as the BR receptor in Arabidopsis.

## Discussion

The use of knock-out and activation tagging mutants has facilitated the characterization of many important genes involved in phytohormone signaling and biosynthetic pathways. In the case of BRs, the characterization of dwarf mutants mainly in Arabidopsis has contributed to our current understanding of BR biology. However, few studies have evaluated BRs in Poaceae, and most advances in these crops have been made in rice and maize [18–21]. The lack of BR-defective mutants is an obstacle in efforts to reveal BR functions in other model plants and at the time this work started, just one BR mutant had been identified in *Brachypodium* [16]. Subsequently, one more mutant defective in BR biosynthesis has emerged [17].

In this report, we sought to contribute to the knowledge of BR biology using Brachypodium as a model plant by measuring the effects of a potent and specific BR inhibitor, Pcz. We demonstrated that Pcz treatments mimic the characteristic dwarf phenotypes of plants deficient in brassinosteroids and that the response is dose-dependent, similar to what was described for Arabidopsis and maize [18]. Treatments with 50 μM Pcz caused severe phenotypes in seedlings, especially in the roots, where we observed a 75% decrease in length compared to the mock treatment, while the reduction in leaf length was almost 34%. The marked effect on roots may be due to the method used to apply Pcz. As we planted the seedlings in vermiculite soil soaked with Pcz solution, the roots were in permanent and direct contact with Pcz, whereas the aerial tissue was not. Still, this method was able to cause not only a decrease in leaf length, but also other phenotypes in the leaves that were similar to the ones described in transgenic rice plants overexpressing the BR negative regulator BIN2 [10].

Although the Pcz-treated leaves initially seemed to develop relatively normally, they later curled, especially towards the top, and exhibited increased width and thickness compared to the mock treatments. Further analyses using cross-sections of leaves could reveal more about the possible effects of Pcz on venation pattern or cell organization in these tissues. Considering the phenotypes obtained using the chemical inhibitor in Brachypodium, we propose that Pcz treatments represent a powerful tool for studying BRs in this model plant. Pcz could be administered to investigate vascular and stomatal development, root growth, cell elongation, and meristematic cell maintenance, among other specific processes in which BR is known to play active roles [22, 23].

The BR biosynthetic genes are primarily regulated at the transcriptional level, and this regulation is mainly mediated by the transcription factor BZR1, with supplemental regulation by other transcription factors. When BR signaling is activated, the dephosphorylated (i.e., active) form of BZR1 binds to the promotor sequence of BR synthetic genes to repress their transcription. This mechanism is known as negative feedback downregulation of BR biosynthetic genes [4, 24]. In this way, BL treatments reduce the expression of *DWF4*, *CPD*, and *BR6ox2* while Pcz application has the opposite effect since it reduce BR levels [18]. Considering this feedback regulation of the biosynthetic genes, we evaluated the transcript levels of our candidate BR-related genes *BdDWF4* (*Bradi1g69040*), *BdCPD* (*Bradi4g43110*), and *BdBR6ox* (*Bradi1g15030*) in seedlings subjected to Pcz treatment. We observed the Pcz-dependent induction of the three candidates tested, although the increase in *BdCPD* expression level was lower than that of the other two genes. However, a similar result was observed previously for Arabidopsis *CPD* upon Pcz application [18], possibly because another mechanism regulates the post-transcriptional level of *CPD*. Taken together, *BdDWF4*, *BdCPD*, and *BdBR6ox* are regulated by a feedback loop at the transcript level once BR levels decrease upon Pcz treatments, suggesting their possible role as enzymes involved in BR synthesis.

To date, many BR genes homologs to the ones characterized from *A. thaliana* have been identified in different monocot species and most of these works are based on the description of the phenotypes from mutant plants which in most of cases present dwarfism or other characteristic BR-related phenotypes. Nevertheless, not many of these studies examine the effects of the ectopic overexpression of these suggested homolog genes in Arabidopsis to examine if they fulfill similar roles to its dicot equivalent. Some exceptions are the overexpression of maize *DWF4* (*ZmDWF4*) and rice *GSK1*, one of the homologs of *BIN2*, in *Arabidopsis* [25, 26]. Ectopic overexpression of *ZmDWF4* generated Arabidopsis plants with longer petioles, narrower leaves, and increased size [25], which is similar to the effects we obtained by overexpressing BdDWF4. This typical growth-promoting phenotype is also portrayed by transgenic plants overexpressing *AtDWF4* [27] and the gain-of-function mutant *gulliver3* - *D*/*dwarf4 – D*, which we used as a control to compare the effects of our overexpression [28]. In case of BIN2, opposite phenotypes are observed due to its role in modulating BZR1/BES1 degradation. Heterologous expression of the rice *BIN2* homolog *OsGSK1* in Arabidopsis resulted in plants that were barely one-third the height of control plants [26]. Similarly, overexpression of *BdBIN2* affected plant height and other features in our experiments, but to a greater extent. This difference may be due to OsGSK1 having a minor role in BR signaling, since another homolog of BIN2 in rice, OsGSK2, seems to be a more prominent negative regulator that functions upstream of transcription factors such as OsBZR1, LIC, and DTL [10].

Homologs of the BR receptor BRI1 have been identified and studied in three monocot species to date, including rice, barley, and Brachypodium [29–32]. The functions of the BRI1 homolog in Brachypodium were revealed in studies of plants in which *BdBRI1* was silence by RNAi technology [30], while the homologs in the other two species were characterized using mutant plants. In addition to OsBRI1, BRI1-LIKE homologs (OsBRL1 and OsBRL3) have also been characterized in rice, and in both rice and barley different alleles of *BRI1* have been found in later works [33, 34]. However, these studies failed to determine if the monocot homologs of BRI1 are able to function as a BR receptor in dicot plants. This fact is indeed important, since the end product of BR synthesis and more active BR in monocots does not seem to be brassinolide, but castasterone. Thus, the structure of BRI1 remained to be compared in monocot and dicot plants [11]. To do so, we first searched the Brachypodium genome for *BRI1* putative homologs. We found that *Bradi2g48280* likely functions as a BR receptor in Brachypodium [30]; however, to test our hypothesis, we cloned the gene and heterologously expressed it in *A. thaliana bri1*-*5* mutant plants. To our surprise, the full-length coding sequence of *BdBRI1* was not able to rescue the dwarf phenotype of *bri1*-*5*, which is a weak allele of *bri1*. This was observed despite the plants were expressing the Brachypodium version of *BRI1* (Additional file 4: Figure S3B*)*. Several transgenic lines showed a slight increase in total plant height, but still presented extremely curled leaves and defects in flowering and seed formation. As far as we know, this is the first time a monocot version of BRI1 has been expressed in *A. thaliana* to evaluate if complementation is possible. Our results represent an early step for comparing the functions and structures of BRI1 between dicot and monocot plants and thus broaden the understanding about BR response in crop plants. BRs have profound effects on several physiological responses such as plant size, fertility and leaves inclination, thus good biotechnological targets to manipulate plant architecture towards desirable agronomic traits. The identification and characterization of BR genes in Brachypodium along with the use of genetic engineering have the potential to enhance crop yield in important crops and even the use of fertilizers could be potentially reduced with less negative environmental effects.

## Conclusion

Our results demonstrated that the use of propiconazole in Brachypodium mimic the dwarf phenotypes of BR-deficient or –insensitive plants, thus Pcz is a potent tool to study BR responses in this model plant. We identified components of the BR biosynthetic pathway DWF4, CPD and BR6ox2, and BRI1, BIN2, BSU1 and BZR1 of the BR signaling pathway in Brachypodium. We showed that *BdDWF4*, *BdCPD*, and *BdBR6ox* are regulated by negative feedback loop at the transcript level and overexpression of *BdDWF4* in Arabidopsis conferred transgenic plants with similar phenotypes to those overexpressing Arabidopsis DWF4. We concluded that *Bradi1g69040*, *Bradi4g43110*, and *Bradi1g15030* (*BdDWF4*, *BdCPD*, and *BdBR6ox* respectively) encode BR biosynthetic genes with functions similar to their homologs in Arabidopsis. Heterologous expression of *Bradi2g32620* (*BdBIN2*) caused dwarf phenotypes in Arabidopsis. We therefore conclude that *Bradi2g32620* likely functions as BR negative regulator in Brachypodium. Furthermore, the *Bradi2g48280* (*BdBRI1*) gene likely functions as a BR receptor in Brachypodium. However, the full-length coding sequence of *BdBRI1* was not able to rescue the dwarf phenotype of *bri1*-*5*, which is a weak allele of *bri1* in Arabidopsis. This results indicate that BdBRI1 and BRI1 may not be functionally equivalent. Further investigations are needed to verify the differences in the BR biology of monocot and dicot plants.

## Methods

### Plant material and growth conditions

*Brachypodium distachyon* ecotype Bd21, a community standard diploid inbred line, was used in this research according to previous description in Hong et al. [35]. Seeds were sterilized and germinated in water-soaked filter paper for 3 days before being transferred to soil. Plants were grown in growth room conditions at 22 °C and a long-day (16 h light, 8 h dark) photoperiod. *Arabidopsis thaliana* wild-type Col-0 and Ws-2 ecotypes or mutant seeds (*gulliver3* - *D* and *bri1*-*5*) were surface-sterilized before being sprinkled on agar-solidified MS (Murashige and Skoog) medium. After three day of stratification at 4 °C, plates were transferred to a growth room and grown under long-day conditions at 22 °C. After 10 days, seedlings were transferred to soil and maintained under the same conditions in a growth room.

### Chemical treatments and morphometric analysis

For all treatments in *Brachypodium*, 3-day-old seedlings were transferred to coarse vermiculite soaked in the indicated concentrations of propiconazole solution or mock (DMSO) for control plants and grown for an additional 7 days. Seedlings were maintained in a growth room under normal long-day conditions, except for those grown under dark conditions, in which plates were covered with aluminum foil to mimic growth under total darkness. Plants were then harvested, photographed, and analyzed using ImageJ software. Leaf length was measured from the root-shoot transition zone to the tip of the main leaf, whereas the length of the main root was used to determine root length. For leaf width, each leaf was measured in the exact middle to make an appropriate comparison. After measurements were taken, leaves were sectioned in the middle and cleared by washing in ethanol (EtOH) solutions. Sections were first washed in 90% EtOH at 37 °C for 3 h. If necessary, the 90% solution was replaced to expedite clearing. Then sections were replaced in 50% solution for 5 minutes.. Once the sections were cleared, they were placed overnight in 1% Safranin O diluted in 50% ethanol, to stain the veins a brilliant red color and facilitate observation under a microscope. A Primo Vert Inverted Microscope (Zeiss) was used to examine and image the samples, and then ImageJ software was used to calculate the total width and other parameters, such as distance between veins.

### Identification of BR-related genes in Brachypodium

Using the UniProt server (http://uniprot.org/blast/), Arabidopsis and rice (*Oryza sativa*) BR-related genes were searched for in the Brachypodium genome by BLAST. For each search, query peptide sequences of well-known Arabidopsis proteins involved in BR signaling, including BRI1, BIN2, BSU1, and BZR1, and synthesis, including DWF4, CPD, and BR6OX2, were used. A multiple sequence alignment (MSA) was performed using the T-Coffee Multiple Sequence Alignment Server (http,//tcoffee.crg.cat/) on the identified sequences with at least 40% identity and previously characterized proteins with equivalent functions in other plant species. BoxShade software (http,//embnet.vital-it.ch/software/BOX_form.html) was used to identify and visualize conserved sequences. A Neighbor-joining phylogenetic tree was built using the obtained MSA to illustrate the relatedness among known proteins and homolog candidates in *B. distachyon* and to select one for further characterization. A list of the BR-related proteins and candidates in *B. distachyon* used for the alignments and the MSAs can be found in (Additional file 3: Table S1).

### RT-PCR and quantitative real-time RT-PCR

For gene expression analysis, total RNA was prepared from Mock- or Pcz-treated *B. distachyon* seedlings using Trizol (Qiagen). RNA (2 μg) was reverse-transcribed using M-MLV enzyme (ELPIS), following the manufacturer’s instructions, to synthesize cDNA, which was used as a template in PCR reactions. For semi-quantitative RT-PCR, the resulting cDNA samples were diluted (1,10) and equal amounts were used to amplify each gene. The level of gene expression was defined using *BdACT7* as a reference gene. Quantitative real-time RT-PCR detection was performed using the Applied Biosystems StepOne Real-Time PCR System and Power SYBR Green PCR Master Mix in combination with designed primers specific for *BdDWF4*, *BdBR6ox2*, and *BdCPD* (Additional file 5: Table S2). The reactions were performed in triplicate and the product was quantified by generating standard curves using serially diluted cDNA mix of all of the samples. Relative expression, representing fold change over the mock control, was normalized for each cDNA sample using *BdACT7* as internal control. As a negative control (NC) we used distilled water in the same amount of the samples. In case of RT-PCR, the conditions were optimized until clear band of expected size was observed in the samples and no band was detected in the NC. In case of quantitative RT-PCR, NC was run in triplicate along with samples and normally gave us an undetermined Cт value or at least higher value than the most diluted sample from our standard curve to prove the accuracy and specificity of our primers in each run. Melting curves for each set of primers were also generated automatically by the StepOne Software (v2.3) from the Applied Biosystems system.

### Cloning of *BdDWF4*, *BdBIN2*, and *BdBRI1*

To express *BdDWF4*, *BdBIN2*, and *BdBRI1* under the cauliflower mosaic virus 35S promoter (35S) in *A. thaliana*, RNA was extracted and cDNA was synthesized from *B. distachyon* 10-days-old seedlings and specific primers (Additional file 5: Table S2) were used to amplify full-length coding sequences (CDSs) of interest. PCR product was purified and cloned using the Invitrogen Gateway entry vector pENTR/SD/D-TOPO in combination with the destination pEarleyGate101 (C-YFP-HA) vector. The constructs were transformed into Arabidopsis using conventional *Agrobacterium*-mediated techniques. For *35S*,*BdDWF4* and *35S*,*BdBIN2*, plants in the Col-0 background were used, whereas for *35S*,*BdBRI1*, the construct was introduced in *bri1*-*5* mutant background. Transgenic seedlings were selected on MS medium supplemented with 50 mg/L BASTA.

## Additional files

Additional file 1: Figure S1. Brachypodium seedlings exhibit dwarfism in response to propiconazole treatment in dark. Morphology of 7-days-old seedlings subjected to the mock treatment and 50 μM Pcz. (PPTX 1022 kb)
Additional file 2: Figure S2. Propiconazole treatment reduces leaves and roots lengths in Brachypodium seedlings. Total lengths of seedlings, leaves and roots after 7 days of exposure to mock (white bars) or 50 μM Pcz (black bars). The graphs represent average value (*n* > 10) and error bars standard deviation. Significant differences among treatments were determined by Student’s *t*-test.***, *P* < 0.0001. (PPTX 45 kb)
Additional file 3: Table S1. List of BR genes in *A. thaliana* and homolog candidates in Brachypodium. (DOCX 17 kb)
Additional file 4: Figure S3. Phenotypes associated with *BdBRI1ox* lines and *BdBRI1* transcript accumulation. (A) Morphology of 5-week-old transgenic plants selected in Basta media along with Ws-2 wild type and *bri1*-*5* mutant as controls. (B) Expression of *BdBRI1* in five of the transgenic plants was confirmed through RT-PCR. *AtUBQ10* was used as an internal loading control. (PPTX 1026 kb)
Additional file 5: Table S2. Primers used in the research. (DOCX 14 kb)

## Abbreviations

BAK1: BRI1-ASSOCIATED RECEPTOR KINASE 1
BIN2: BR INSENSITIVE 2
BL: Brassinolide
BR: Brassinosteroid
BR6ox2: BRASSINOSTEROID-6-OXIDASE 2
BRI1: BRASSINOSTEROID-INSENSITIVE 1
BZR1: BRASSINAZOLE RESISTANT 1
CPD: CONSTITUTIVE PHOTOMORPHOGENIC DWARF
CS: Castasterone
DTL: DWARF AND LOW-TILLERING
DWF4: DWARF 4
GSK: GSK3/SHAGGY-like kinase
LIC: TILLER ANGLE INCREASED CONTROLLER
Pcz: Propiconazole
TUD1: TAIHU DWARF 1, GA, gibberellin, BRD1, BRASSINOSTEROID DEFICIENT DWARF 1
