# Supplementary figures and images for: Identification of brassinosteroid genes in Brachypodium distachyon
Source: BMC Plant Biol. 2017 Jan 6;17:5. doi: 10.1186/s12870-016-0965-3 (PMC5217202; doi:10.1186/s12870-016-0965-3)

## Slide 1
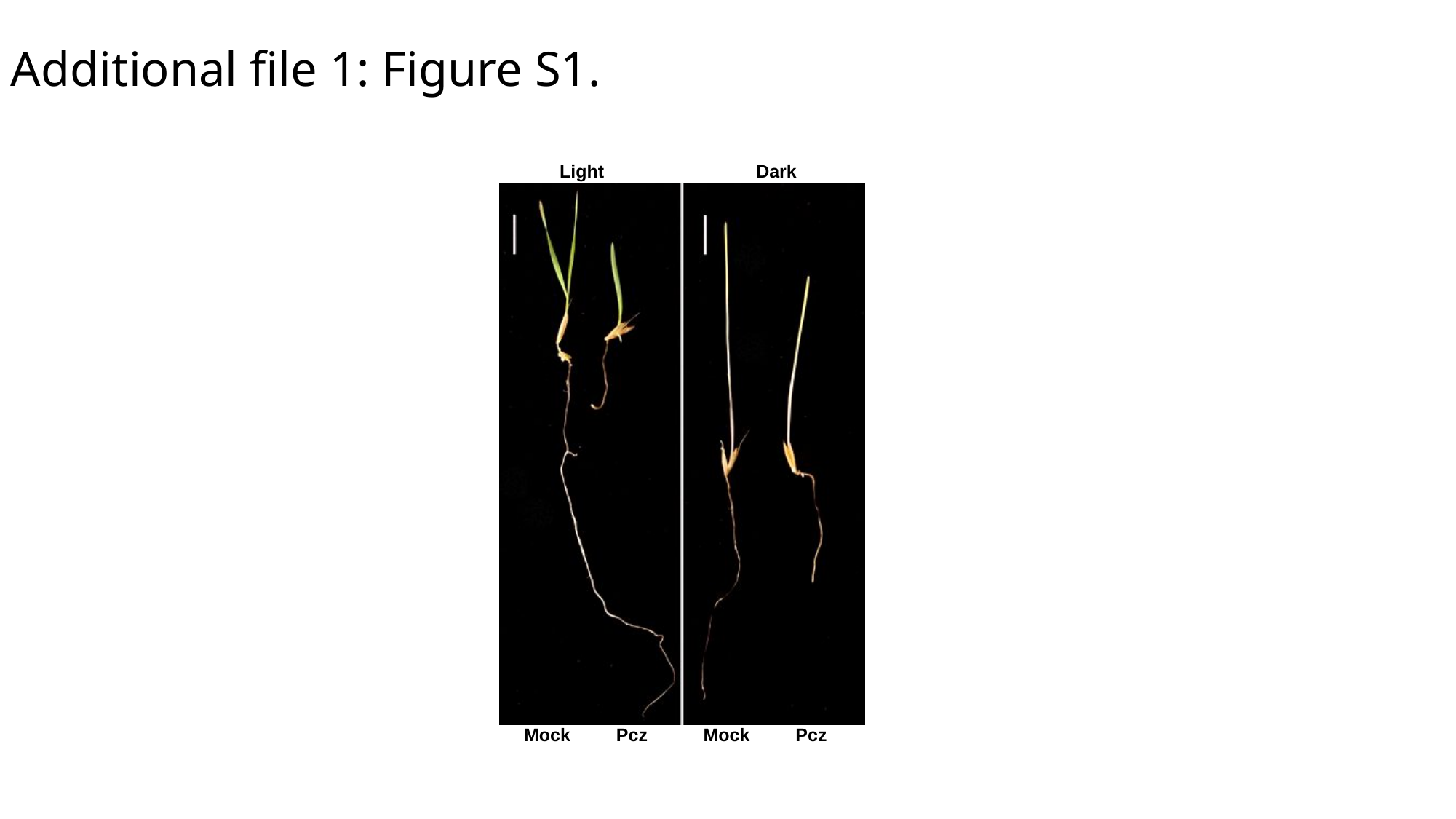

Additional file 1: Figure S1.
 Light Dark
Mock Pcz Mock Pcz

Supplement: Additional file 1: Figure S1. — Brachypodium seedlings exhibit dwarfism in response to propiconazole treatment in dark. Morphology of 7-days-old seedlings subjected to the mock treatment and 50 μM Pcz. (PPTX 1022 kb) [file 12870_2016_965_MOESM1_ESM.pptx]

## Slide 1
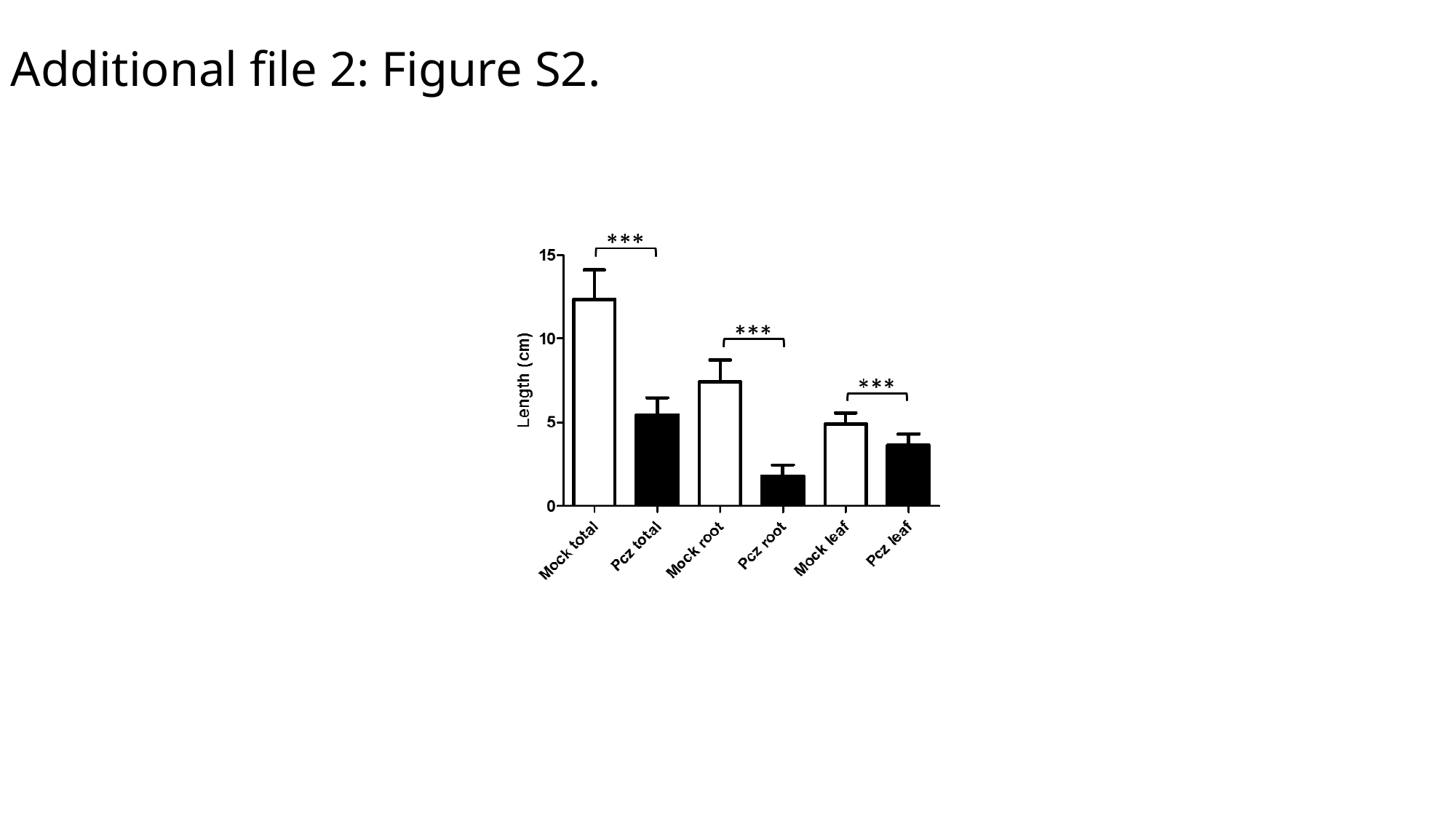

Additional file 2: Figure S2.

Supplement: Additional file 2: Figure S2. — Propiconazole treatment reduces leaves and roots lengths in Brachypodium seedlings. Total lengths of seedlings, leaves and roots after 7 days of exposure to mock (white bars) or 50 μM Pcz (black bars). The graphs represent average value (n > 10) and error bars standard deviation. Significant differences among treatments were determined by Student’s t-test.***, P < 0.0001. (PPTX 45 kb) [file 12870_2016_965_MOESM2_ESM.pptx]

## Slide 1
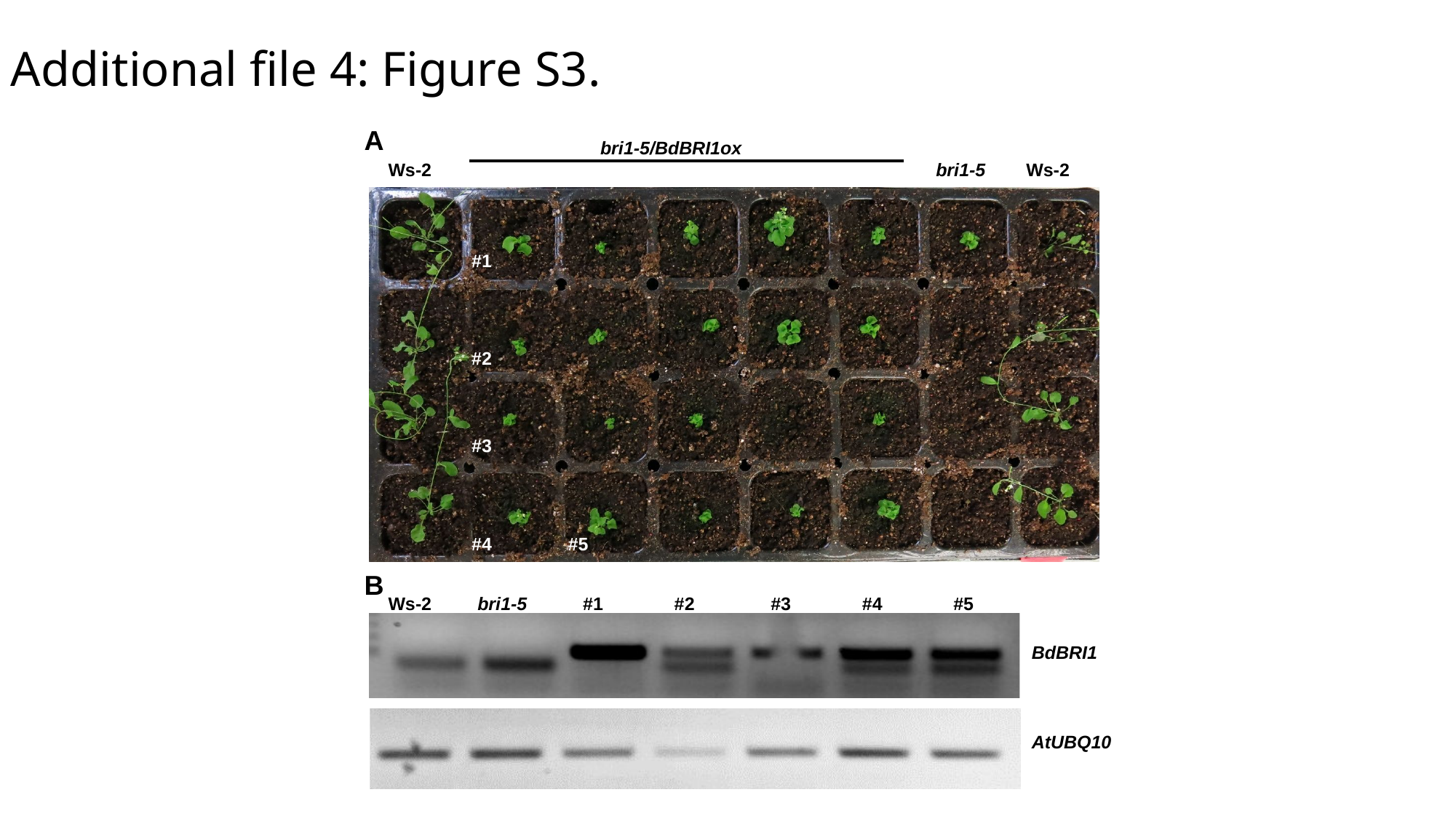

Additional file 4: Figure S3.
A
B
bri1-5/BdBRI1ox
Ws-2 bri1-5 Ws-2
 #1
 #2
 #3
 #4 #5
Ws-2 bri1-5 #1 #2 #3 #4 #5
BdBRI1
AtUBQ10

Supplement: Additional file 4: Figure S3. — Phenotypes associated with BdBRI1ox lines and BdBRI1 transcript accumulation. (A) Morphology of 5-week-old transgenic plants selected in Basta media along with Ws-2 wild type and bri1-5 mutant as controls. (B) Expression of BdBRI1 in five of the transgenic plants was confirmed through RT-PCR. AtUBQ10 was used as an internal loading control. (PPTX 1026 kb) [file 12870_2016_965_MOESM4_ESM.pptx]
