# Supplementary material for: Identification of brassinosteroid genes in Brachypodium distachyon
Source: BMC Plant Biol. 2017 Jan 6;17:5. doi: 10.1186/s12870-016-0965-3 (PMC5217202; doi:10.1186/s12870-016-0965-3)
Supplement: Additional file 3: Table S1. — List of BR genes in A. thaliana and homolog candidates in Brachypodium. (DOCX 17 kb) [file 12870_2016_965_MOESM3_ESM.docx]

|  |  | ***A.thaliana*** | | ***B. distachyon*** | | | | |
| --- | --- | --- | --- | --- | --- | --- | --- | --- |
| **Gene** | **Description** | **Gene ID** | **Protein ID** | **Candidate Homologs** | **Candidate protein ID** | **Identity** | **Score** | **E-value** |
| BRI1 | BR signaling (BR receptor) | *At4g39400* | O22476 | *Bradi2g48280* | I1HQZ9 | 53.6% | 2971 | 0.0 |
|  |  |  |  | *Bradi4g27440* | I1IP60 | 50.8% | 2572 | 0.0 |
|  |  |  |  | *Bradi3g21400* | I1I325 | 45.6% | 2122 | 0.0 |
| BIN2 | BR signaling (negative regulator) | *At4g18710* | Q39011 | *Bradi2g32620* | I1HKY2 | 86.3% | 1740 | 0.0 |
|  |  |  |  | *Bradi2g06490* | I1HKY0 | 89.0% | 1731 | 0.0 |
|  |  |  |  | *Bradi3g09067* | I1HD48 | 82.5% | 1611 | 0.0 |
| BSU1 | BR signaling (positive regulator) | *At1g03445* | Q9LR78 | *Bradi2g36370* | [I1HM01](http://www.uniprot.org/uniprot/I1HM01) | 51.6% | 2183 | 0.0 |
|  |  |  |  | *Bradi1g13710* | I1GQ04 | 43.9% | 1869 | 0.0 |
|  |  |  |  | *Bradi4g01410* | I1IGC2 | 37.5% | 1399 | 8.30E-177 |
| BZR1 | BR signaling (positive regulator) | *At1g75080* | Q8S307 | *Bradi1g23550* | I1GT13 | 52.5% | 799 | 7.10E-102 |
|  |  |  |  | *Bradi2g06400* | I1HD37 | 41.0% | 523 | 1.40E-60 |
|  |  |  |  | *Bradi1g38180* | I1GY36 | 40.8% | 517 | 1.10E-59 |
| DWF4 (CYP90B1) | BR biosynthesis | *At3g50660* | O64989 | *Bradi1g69040* | I1H7R8 | 66.5% | 1733 | 0.0 |
|  |  |  |  | *Bradi5g12990* | I1IYP5 | 42.4% | 1035 | 6.50E-133 |
| CPD (CYP90A1) | BR biosynthesis | *At5g05690* | Q42569 | *Bradi4g43110* | I1IUJ6 | 62.2% | 1415 | 0.0 |
|  |  |  |  | *Bradi2g05980* | I1HCZ1 | 41.2% | 919 | 9.10E-116 |
| BR6OX2 (CYP85A2) | BR biosynthesis | *At3g30180* | Q940V4 | *Bradi1g15030* | I1GQE7 | 60.9% | 1524 | 0.0 |

Additional file 3: Table S1

**Table S1.**

List of BR genes in *A. thaliana* and homolog candidates in Brachypodium.
