# Supplementary material for: Identification of brassinosteroid genes in Brachypodium distachyon
Source: BMC Plant Biol. 2017 Jan 6;17:5. doi: 10.1186/s12870-016-0965-3 (PMC5217202; doi:10.1186/s12870-016-0965-3)
Supplement: Additional file 5: Table S2. — Primers used in the research. (DOCX 14 kb) [file 12870_2016_965_MOESM5_ESM.docx]

| Gene | Locus ID | Direction | Sequence (5' to 3') | Size (bp) | Purpose |
| --- | --- | --- | --- | --- | --- |
| *BdDWF4* | *Bradi1g69040* | forward | **CACC**ATGGCCGCCATGATGGCGTCCATAACC | 1521 | cloning |
|  |  | reverse | TATATCCACGGTCAAAACACTTTTCTCTTC |  |  |
| *BdBRI1* | *Bradi2g48280* | forward | **CACC**ATGGATTCCTTGCGGGTGGCGATAGC | 3369 | cloning |
|  |  | reverse | ATCCTTCTCTTCCTTGGCTTCCTTGAGG |  |  |
| *BdBIN2* | *Bradi2g32620* | forward | **CACC**ATGGAGCAGACGGCGCCAGCGGCG | 1215 | cloning |
|  |  | reverse | GCTCCCAGCATGGAAGAAGTTGAAGCCG |  |  |
| *BdACT 7* | *Bradi4g41850* | forward | TCCTTTTCCAGCCATCTTTC | 328 | RT-PCR |
|  |  | reverse | ATCGCTGGACCAGACTCATC |  |  |
| *BdDWF4* | *Bradi1g69040* | forward | GCACCGGAAAGTAATTCGAG | 331 | RT-PCR |
|  |  | reverse | CTTTGGAAAGTCCACGAAGG |  |  |
| *Bd85A2* | *Bradi1g15030* | forward | ATGGCCTTGCTATCTGCACT | 368 | RT-PCR |
|  |  | reverse | ACAGGTACTTGACCGCCATC |  |  |
| *BdCPD* | *Bradi4g43110* | forward | TCAATCTCACCGTCAAGCAG | 339 | RT-PCR |
|  |  | reverse | ACAGGCAGAAGTCCACCATC |  |  |
| *BdGA20ox* | *Bradi2g24980* | forward | ACGGCTTCTTCCTGGTGAC | 553 | RT-PCR |
|  |  | reverse | CGCGAAGAAGTCCCTGTAGT |  |  |
| *Bd85A2* | *Bradi1g15030* | forward | AGCGGAAATTGCAACGTTCC | 121 | real-time RT-PCR |
|  |  | reverse | AACTCGGATATGTAGCCCGTTG |  |  |
| *BdCPD* | *Bradi4g43110* | forward | TTTCGCTTCATTCCGAGCTG | 91 | real-time RT-PCR |
|  |  | reverse | TTTGTGGTTGCTCTGCCATC |  |  |
| *BdDWF4* | *Bradi1g69040* | forward | AGATGGAAGGGCAATGCATC | 125 | real-time RT-PCR |
|  |  | reverse | AGGTGGTGCAAAAAGATGGC |  |  |
| *BdBIN2* | *Bradi2g32620* | forward | CAATAGATCTTGCTTCGCGG | 146 | real-time RT-PCR |
|  |  | reverse | GTTGAATAGAGGTGGGAATGGG |  |  |
| *BdBRI1* | *Bradi2g48280* | forward | TCTCTTTCAACCACTTCACCG | 137 | real-time RT-PCR |
|  |  | reverse | GAGACTGGAGTTGGGATCTTG |  |  |
| *AtUBQ10* | *AT4G05320* | forward | TCCAGGACAAGGAAGGTATTCC | 205 | real-time RT-PCR |
|  |  | reverse | CCACCAAAGTTTTACATGAAACGAA |  |  |

Additional file 5: Table S2

**Table S2.**
Primers used in the researchs
